# A novel pyroptosis-related gene signature to predict outcomes in laryngeal squamous cell carcinoma

**DOI:** 10.18632/aging.203783

**Published:** 2021-12-15

**Authors:** Chongchang Zhou, Guowen Zhan, Yangli Jin, Jianneng Chen, Zhisen Shen, Yi Shen, Hongxia Deng

**Affiliations:** 1 Department of Otorhinolaryngology Head and Neck Surgery, Ningbo Medical Center Lihuili Hospital, Ningbo 315040, Zhejiang, China; 2 Department of Otorhinolaryngology Head and Neck Surgery, Lihuili Hospital Affiliated to Ningbo University, Ningbo 315040, Zhejiang, China; 3 Department of Otorhinolaryngology Head and Neck Surgery, Ningbo Yinzhou Second Hospital, Ningbo 315040, Zhejiang, China; 4 Department of Ultrasonography, Ningbo Yinzhou Second Hospital, Ningbo 315040, Zhejiang, China; 5 Department of Otorhinolaryngology Head and Neck Surgery, Ningbo Zhenhai Longsai Hospital, Ningbo 315200, Zhejiang, China

**Keywords:** pyroptosis, outcome, LSCC, immune microenvironment, immunotherapy

## Abstract

Pyroptosis, a pro-inflammatory form of programmed cell death, is associated with carcinogenesis and progression. However, there is little information concerning pyroptosis-related genes (PRGs) in laryngeal squamous cell carcinoma (LSCC). Herein, we aim to explore the prognostic value of PRGs in LSCC. The expression and clinical data of 47 PRGs in LSCC patients were obtained from The Cancer Genome Atlas. A novel prognostic PRG signature was constructed using least absolute shrinkage and selection operator analysis. Receiver operating characteristic (ROC) curves were drawn, and Kaplan-Meier survival Cox proportional hazard regression analyses were performed to measure the predictive capacity of the PRG signature. Furthermore, we constructed a six-PRG signature to divide LSCC patients into high- and low-risk groups. Patients in the high-risk group had worse overall survival than the low-risk group. The area under the time-dependent ROC curve was 0.696 for 1 year, 0.784 for 3 years, and 0.738 for 5 years. We proved that the PRGs signature was an independent predictor for LSCC. Functional enrichment analysis indicated that several immune-related pathways were significantly enriched in the low-risk group. Consistent with this, patients with low-risk scores had higher immune scores and better immunotherapeutic responses than the high-risk group. In conclusion, we established a novel PRGs signature that can predict outcome and response to immunotherapy of LSCC, pyroptosis may be a potential target for LSCC.

## INTRODUCTION

Laryngeal cancer is the second most common malignant tumor of the head and neck, of which laryngeal squamous cell carcinoma (LSCC) is the most prominent pathological type. LSCC is responsible for ~ 98% of all laryngeal cancers [1]. Etiological factors for LSCC are diverse and include genetic background, environmental factors, alcohol consumption, cigarette smoking, and viral infections [2]. Approximately 1.8 million new cases and 1 million deaths of LSCC occur worldwide per year [3]. Although the therapeutic strategies for LSCC have progressed in recent decades, the 5-year survival rate remained 40% in patients with metastatic and recurrent disease [4], owing to the lack of nonspecific symptoms and effective methods for early diagnosis. Predicting outcomes with high accuracy will potentially improve outcomes and direct individualized treatment. Therefore, the identification of novel and reliable prognostic signatures for LSCC is urgently required.

Pyroptosis is an inflammatory and programmed cell death triggered by cytosolic sensing of invasive infection and other stimuli. Morphologically, pyroptotic cells are characterized by cellular swelling, bubble-like protrusions, and pore formation in the cell membrane by gasdermin (GSDM) family (including GSDMA, GSDMB, GSDMC, GSDMD, and GSDME). These phenomena result in rapid cell death, dying cells appear to flatten as the cytoplasmic contents and interleukins (IL) are released [5]. Pyroptosis is usually but not always results from inflammatory caspase activation via classical and non-classical pathways. In caspase-1-dependent classical inflammasomes, nuclear factor of κB (NF-κB) or tumor necrosis factor and IL-1β bind to corresponding intracellular receptors after cells are stimulated by pathogen-associated and damage-associated molecular patterns or other immune stimulations. Nod-like receptors (NLR, including NLRP1, NLRP2, NLRP3, NLRP6, NLRP7, and NLRC4) and absent in melanoma 2 can be selectively activated, which leads to cleavage of pro-caspase-1 to form activated caspase-1 [6]. In caspase-4/5-dependent non-classical inflammasomes, pro-caspase-4/5 is activated by cytosolic bacterial lipopolysaccharide from invading gram-negative bacteria in macrophages and other cells [6]. Activated caspase-1/4/5 cleaves GSDMD and forms an N-terminal GSDMD fragment that creates a pore in the membrane and causes pyroptosis [7, 8]. Caspase-3 is cleaved by Asp270, which converts cells that undergo GSDME noninflammatory apoptotic death into those that undergo inflammatory pyroptotic death [9, 10].

In some instances (especially cancer), human health improves with cell death. Pyroptosis has been shown to play an essential role in regulating carcinogenic processes, suggesting its potential for cancer therapy and outcome prediction [11, 12]. Pyroptosis is closely linked to the development and progression of gastric cancer [13], breast cancer [14], esophageal carcinoma [15], lung cancer [16], and colorectal cancer [17]. Nevertheless, the association between pyroptosis-related genes (PRGs) and outcome in LSCC remains unclear.

In the current study, we performed a systematic analysis to measure expression characteristics and prognostic values of PRGs in LSCC patients from The Cancer Genome Atlas (TCGA). Then, we constructed a PRG signature to calculate the risk score for predicting overall survival and evaluate the biological function in high- and low-risk patients to explore the potential mechanisms. Finally, the correlation between PRG signature and tumor immune microenvironment, immune infiltration, immunotherapy response, and chemosensitivity were analyzed to identify potential strategies for targeted treatment of LSCC.

## RESULTS

### The expression landscape of PRGs in LSCC patients

**Figure 1:**
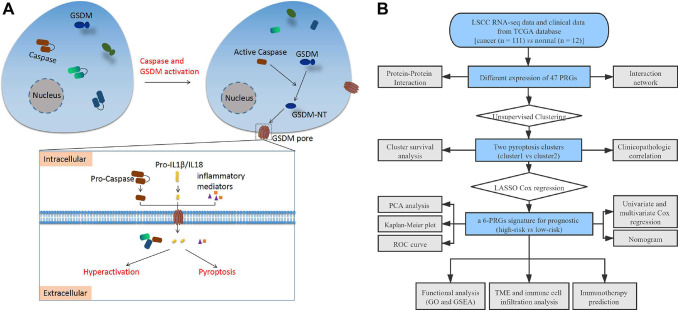
(**A**) Schematic diagram of pyroptosis. (**B**) The flow chart of this study. Abbreviations: LSCC: laryngeal squamous cell carcinoma; TCGA: the cancer genome atlas; PRGs: pyroptosis-related genes; LASSO: least absolute shrinkage and selection operator; PCA: principal component analysis; ROC: receiver operating characteristic; TME: tumor microenvironment; GO: Gene Ontology; GSEA: Gene set enrichment analysis.

**Figure 2:**
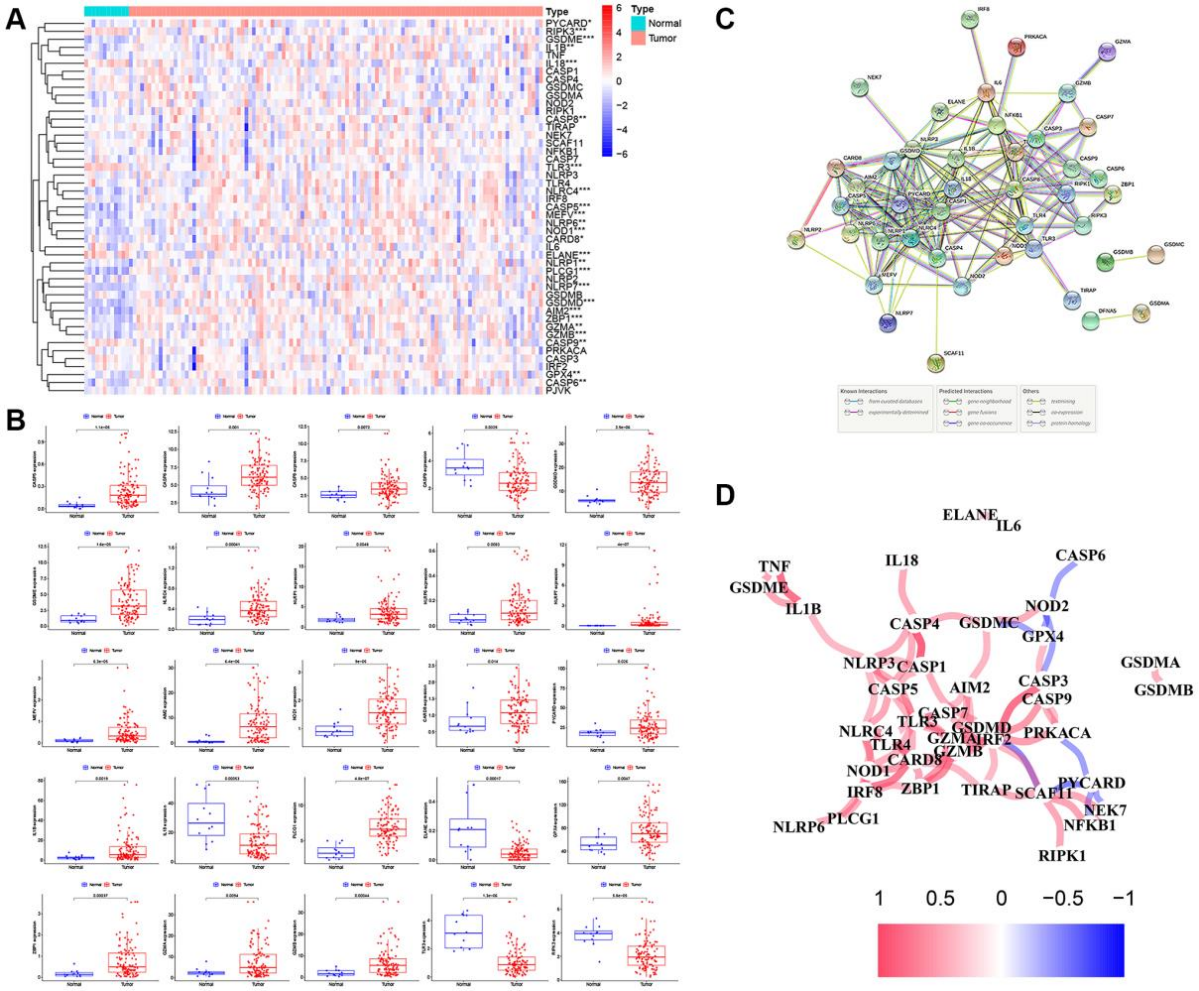
**The expression landscape of PRGs in LSCC patients.** (**A**) The heatmap of the 47 PRGs between LSCC tissues and adjacent normal tissues. *P*-values are as follows: ^*^P < 0.05; ^**^P < 0.01; ^***^P < 0.001. (**B**) The boxplots of differential expression of PRGs between LSCC samples and normal tissues. (**C**) The PPI network of the PRGs using the STRING database. (**D**) The correlation network of the PRGs (red line: positive correlation; blue line: negative correlation. The depth of the colors represents the degrees of correlation).

The methods used for this study are summarized in the flow chart, as shown in Figure 1B. The expression profile of 47 PRGs was compared between 111 LSCC tissues and 12 adjacent normal tissues obtained from TCGA. As presented with a heatmap in Figure 2A (red: high expression level; blue: low expression level), we identified 25 PRGs that were significantly differentially expressed in LSCC patients (all *P* < 0.05). Of these, 20 genes were significantly up-regulated in the tumor samples, while 5 genes were down-regulated (Figure 2B). We performed a PPI analysis to explore the interconnections of PRGs, and found that they were highly connected, especially *CARD8, GSDMD, TLR3,* and *CASP1* (Figure 2C). Consistent with this, the expression correlation network of the PRGs in LSCC is shown in Figure 2D. The depth of the colors represents the strength of the correlation.

### Pyroptosis clusters based on differentially expressed PRGs

**Figure 3:**
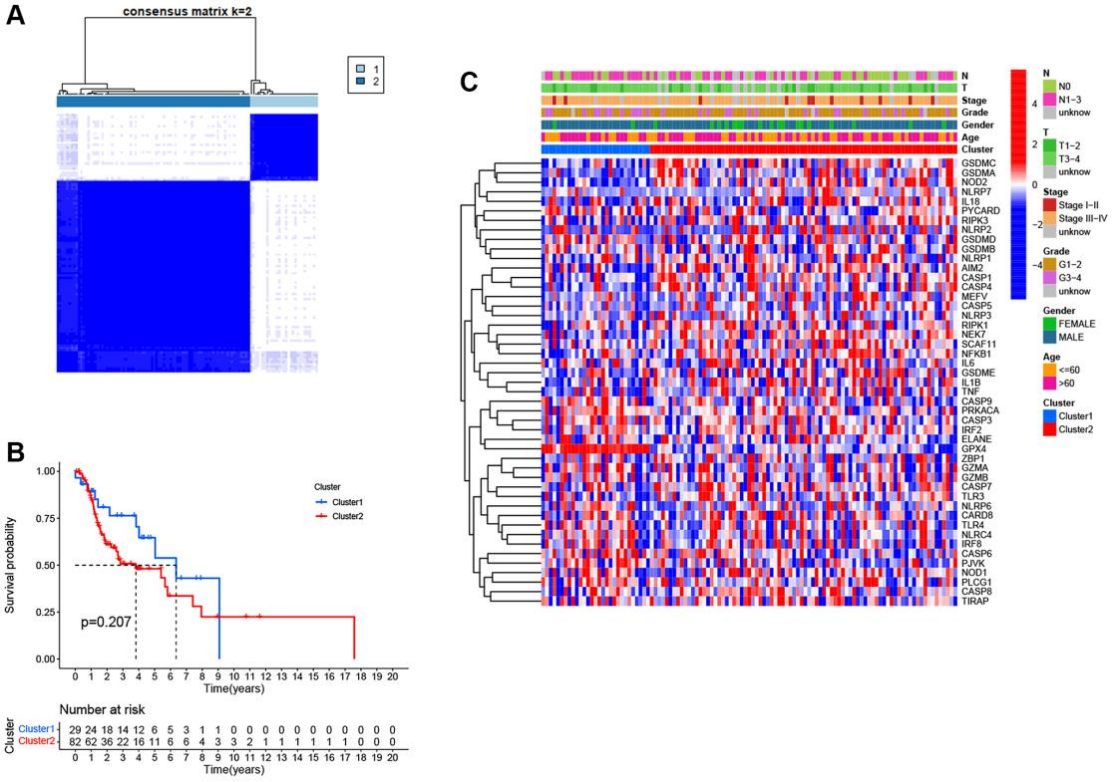
**Pyroptosis clusters based on the differentially expressed PRGs.** (**A**) Consensus clustering of 111 LSCC samples when *k* = 2. (**B**) Kaplan-Meier survival analysis with the log-rank test between the two clusters (*P* = 0.207). (**C**) Heatmap of clinicopathological characteristics and clusters.

To identify distinct pyroptosis-related patterns, we utilized a consensus clustering analysis with all 111 LSCC patients from TCGA based on the differential expression of 25 PRGs. The clustering variable *k* = 2 was determined to be an optimal clustering stability from *k* = 2 to 9, indicating that 111 LSCC patients could be classified into two clusters, cluster 1 (*n* = 29) and cluster 2 (*n* = 82) (Figure 3A). Kaplan-Meier survival analysis with log-rank test was used to compare the overall survival between two clusters. However, we found that the patients in cluster 2 tended to have worse outcomes than cluster 1, without a statistically significant difference (Figure 3B, *P* = 0.207). The relationship between the two clusters and clinical factors, including age, gender, histologic grade, T stage, N stage, and clinical stage, were drawn in a heatmap (Figure 3C). However, there were no differences in clinical features between the two clusters.

### Construction of the PRG signature for LSCC patients

**Figure 4:**
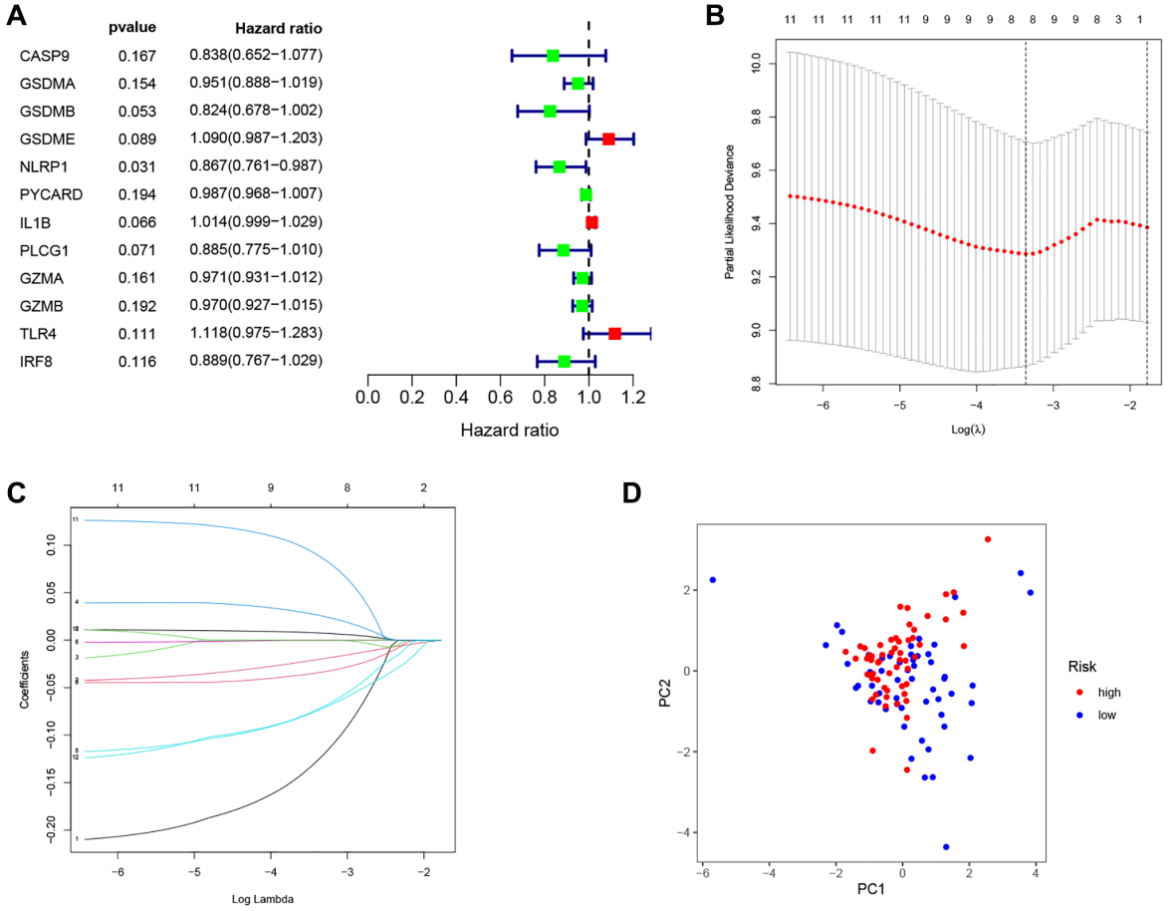
**Construction of the PRG-based signature for LSCC patients.** (**A**) The prognostic PRGs using univariate Cox regression analysis (*P* < 0.2). (**B**) Optimal parameter (λ) selected in the LASSO Cox regression model based on the minimum criteria. (**C**) The LASSO coefficient of the pyroptosis-related signature. (**D**) Score plot for the PCA analysis.

As shown in Figure 4A, 12 prognostic PRGs (*CASP9, GSDMA, GSDMB, GSDME, NLRP1, PYCARD, IL1B, PLCG1, GZMA, GZMB, TLR4* and *IRF8*) were identified using univariate Cox regression analysis. Six PRGs (*CASP9*, *GSDMA*, *NLRP1*, *IL1B*, *TLR4*, and *IRF8*) were selected to construct the PRG signature according to optimal parameter (λ) of the LASSO regression analysis (Figure 4B, 4C). The risk score for the total sample was then calculated follows: risk score = (−0.208 × *CASP9*) + (−0.047 × *GSDMA*) + (−0.116 × *NLRP1*) + (0.012 × *IL1B*) + (0.131 × *TLR4*) + (−0.149 × *IRF8*). We divided 111 LSCC patients into high-risk (*n* = 56) and low-risk groups (*n* = 55) using the median risk score as the cut off value. The PCA plot showed the high- and low-risk groups were distinct (Figure 4D).

### Validation of the prognostic value of the PRG signature for LSCC

**Figure 5:**
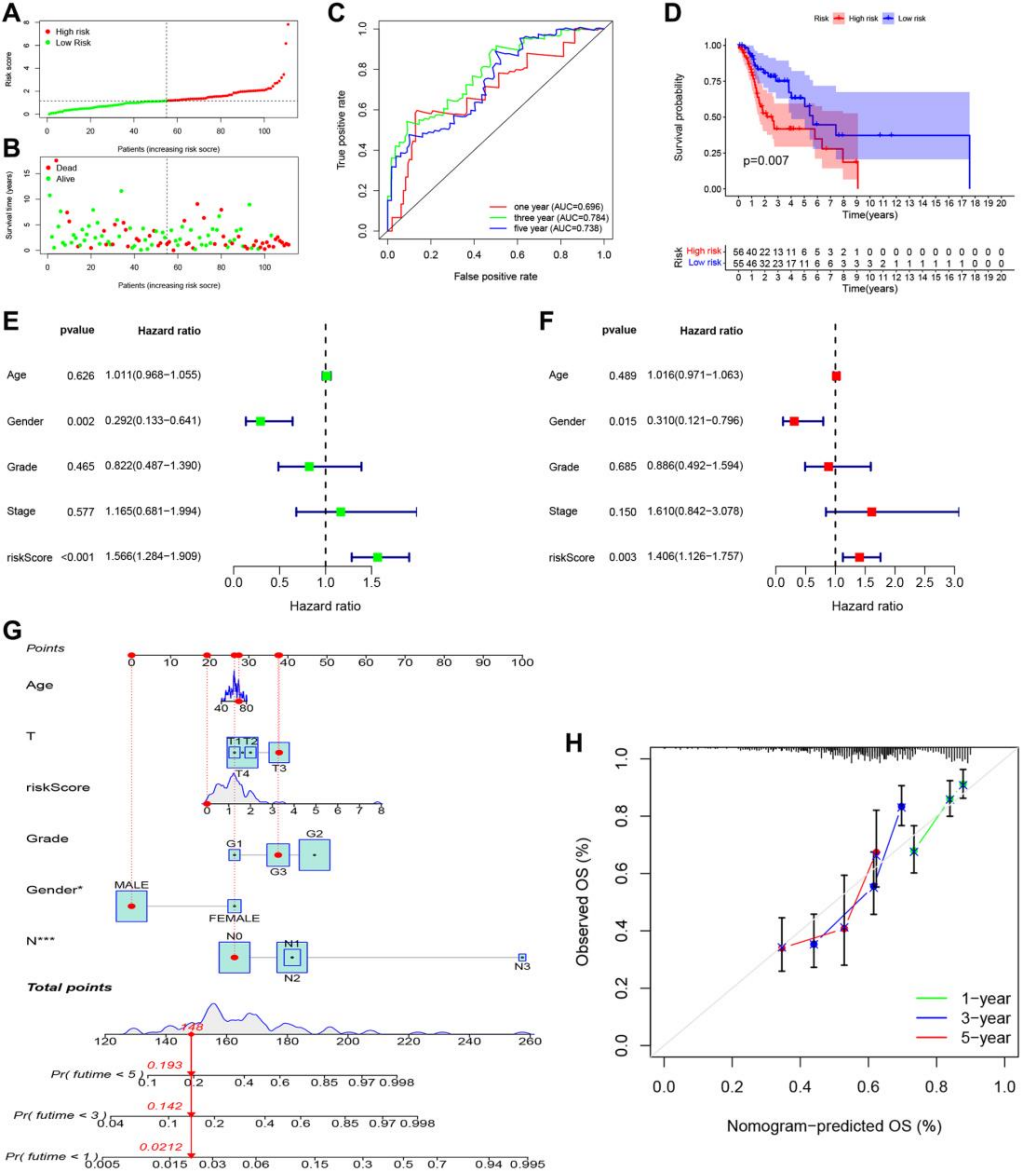
**Independent prognostic value of PRGs-based signature.** (**A**) The distribution and median value of the risk scores. (**B**) The distributions of overall survival status, overall survival, and risk score. (**C**) The AUC values of time-dependent ROC curves for survival prediction. (**D**) Kaplan-Meier survival curves showing the overall survival of high- and low-risk LSCC patients divided according to the risk score (log-rank *P* = 0.007). (**E**) Prognostic value of the risk scores in the univariate Cox regression analysis (HR = 1.566, 95% CI: 1.284–1.909, *P* < 0.001). (**F**) Prognostic value of the risk scores in the multivariate Cox regression analysis (HR = 1.406, 95% CI: 1.126–1.757, *P* = 0.003). (**G**) The nomogram for predicting the 1-year, 3-year, and 5-year overall survival. (**H**) Calibration plot of the nomogram for predicting 1-year, 3-year, and 5-year overall survival.

As shown in Figure 5A, 5B, patients in the high-risk group had a shorter survival time and a higher probability of death than those in the low-risk group. Moreover, the time-dependent ROC curve showed the AUC reached 0.696 for 1 year, 0.784 for 3 years, and 0.738 for 5 years (Figure 5C). Kaplan-Meier survival analysis showed that the high-risk group had significantly worse overall survival than the low-risk group (Figure 5D, *P* = 0.007). Univariate (Figure 5E; HR = 1.566, 95% CI: 1.284–1.909, *P* < 0.001) and multivariate (Figure 5F; HR = 1.406, 95% CI: 1.126–1.757, *P* = 0.003) Cox regression indicated that the risk score was an independent prognostic factor of overall survival for LSCC. We then built a nomogram to predict 1-year, 3-year, and 5-year overall survival (Figure 5G). The calibration plot indicated that the nomogram had good predictive performance and accuracy compared to the ideal model (Figure 5H). Next, we analyzed the relationship between the clinical factors and risk score by the Chi-square test and Wilcoxon signed-rank test. The heatmap (Supplementary Figure 1A) and the scatter plots showed that there was no significantly correlation in age (Supplementary Figure 1B), gender (Supplementary Figure 1C), tumor grade (Supplementary Figure 1D), clinical stage (Supplementary Figure 1E), T classification (Supplementary Figure 1F), and lymph node metastasis (Supplementary Figure 1G).

### Functional enrichment analysis

**Figure 6:**
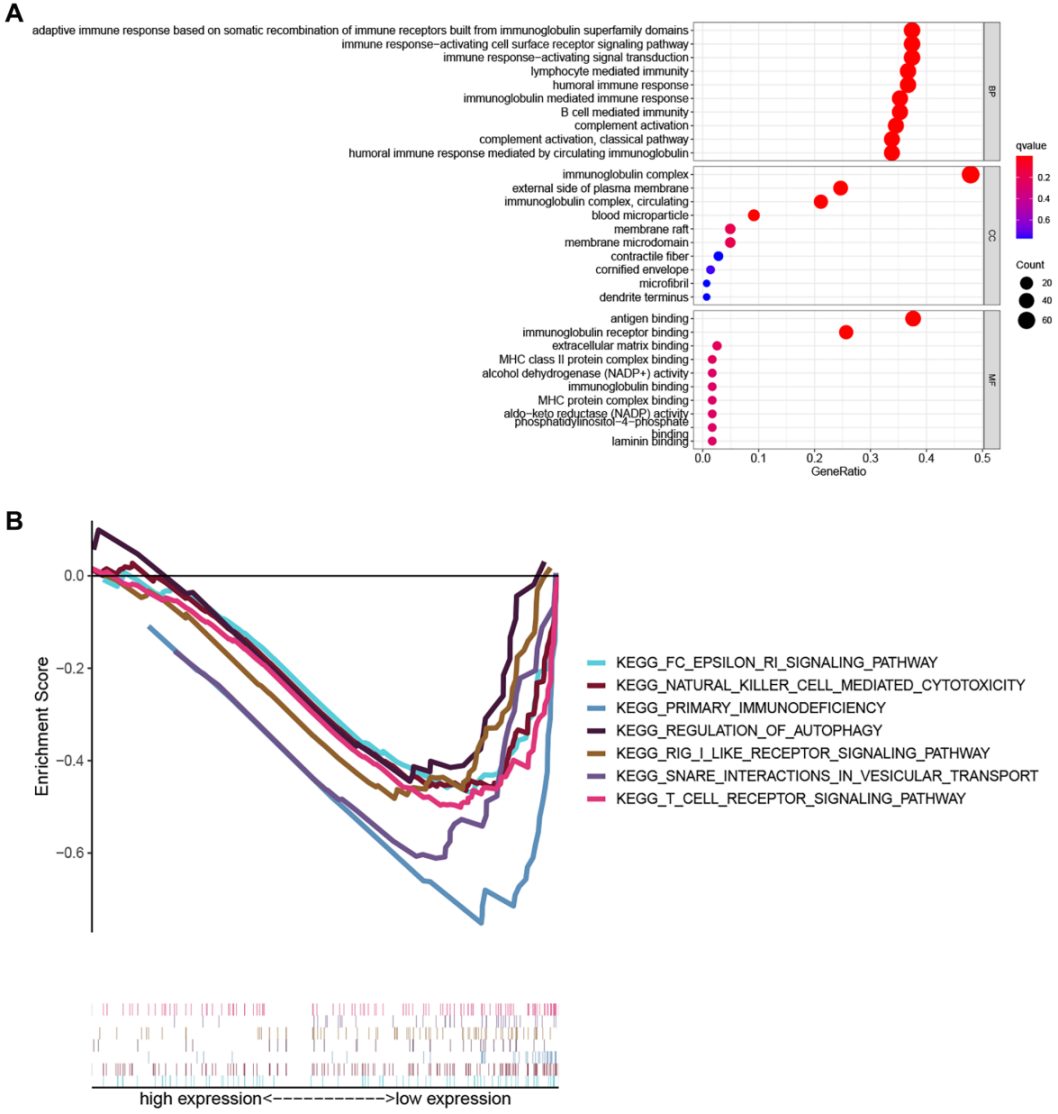
**Functional enrichment analysis.** (**A**) The top ten biological process (BP) terms, cellular components (CC) terms, molecular functions (MF) terms of GO analysis. (**B**) GSEA analysis showing seven pathways enriched in the low-risk group.

To explore the biological functions and pathways related to the PRG signature, GO enrichment and GSEA analyses were performed based on the DEGs between the high- and low-risk groups. As shown in Figure 6A, the results of GO analysis indicated that DEGs were involved in immune-related biological processes such as adaptive immune response based on somatic recombination of immune receptors built from immunoglobulin super family domains, the immune response-activating cell surface receptor signaling pathway, immune response-activating signal transduction, lymphocyte-mediated immunity, humoral immune response, immunoglobulin mediated immune response, and B cell-mediated immunity. Consistently, the GSEA analysis revealed that several immune-related and cancer-related pathways were significantly enriched in the low-risk group, including primary immunodeficiency, the Fc epsilon RI signaling pathway, natural killer cell-mediated cytotoxicity, and the T cell receptor signaling pathway (Figure 6B).

### The relationship between the PRG signature and immune status

**Figure 7:**
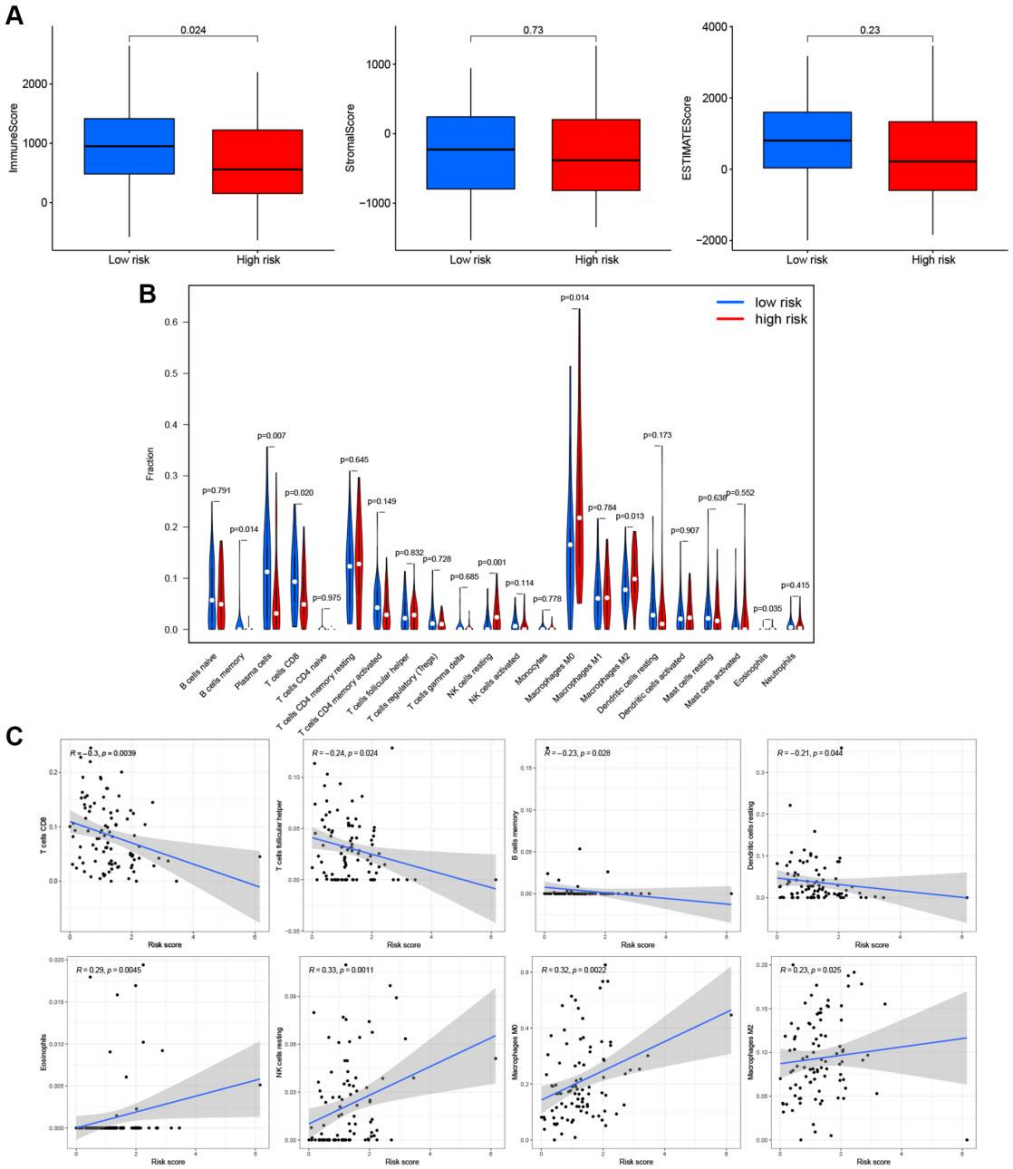
**Effects of PRGs-based prognostic model on immune cell infiltration.** (**A**) Comparison of the immune, stromal, and estimate scores in the low- and high-risk groups, respectively. (**B**) The violin plot of different infiltration levels of immune cells between high- and low-risk patients. (**C**) The correlation of risk score and immune cells infiltration.

Based on these findings, the PRG signature appeared to be closely related to immune processes. We assessed the effects of the PRGs-based prognostic model on the LSCC tumor immune microenvironment. LSCC patient in the low-risk group had higher immune scores (*P* = 0.024), stromal scores (*P* = 0.73) and estimate scores (*P* = 0.23) than the high-risk group (Figure 7A), although the *P*-values were <0.05 only for the immune score. This finding suggests that the low-risk group might have more immune cells than the high-risk group.

**Figure 8:**
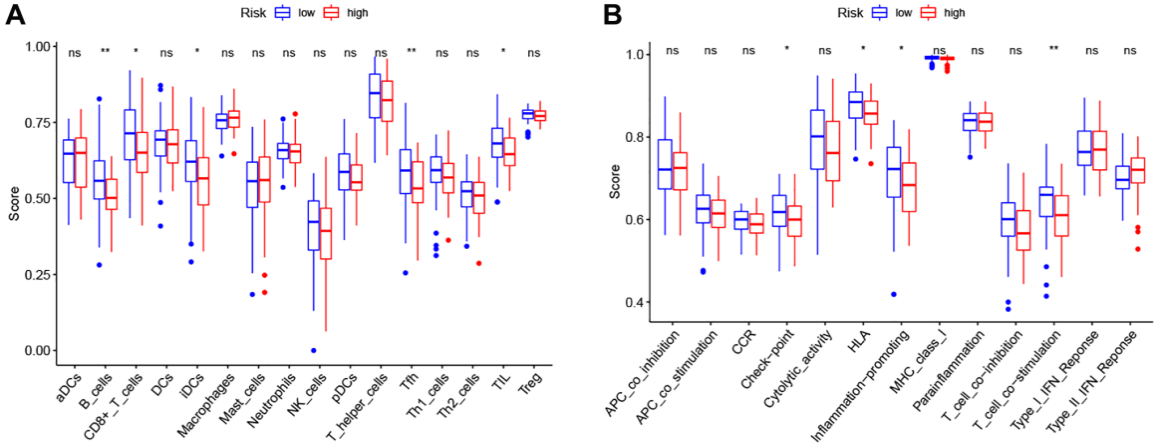
**Comparison of the ssGSEA scores between high- and low-risk groups.** (**A**) The scores of 16 immune cells. (**B**) The scores of 13 immune-related functions. Adjusted *P*-values were showed as: ns, not significant; ^*^P < 0.05; ^**^P < 0.01; ^***^P < 0.001.

Therefore, we further analyzed the connection between the PRG-based prognostic model and immune cells infiltration. As expected, the low-risk group generally had higher infiltration of memory B cells, plasma cells, and CD8+ T cells and lower infiltration of resting NK cells, M0 macrophages, M2 macrophages, and eosinophils (Figure 7B). Correlation analysis indicated that CD8+ T cells, T cells follicular helper cells (Tfh), memory B cells, and resting dendritic cells had strong negative correlations with risk scores (Figure 7C). Resting NK cells, M0 macrophages, M2 macrophages, and eosinophils positively correlated with risk score (Figure 7C). Finally, ssGSEA analysis showed that checkpoint, HLA, inflammation-promotion, and T cell co-inhibition immune pathways were more highly activated in LSCC patients with low-risk scores (Figure 8A, 8B).

### Immunotherapy prediction of PRG signature for LSCC

**Figure 9:**
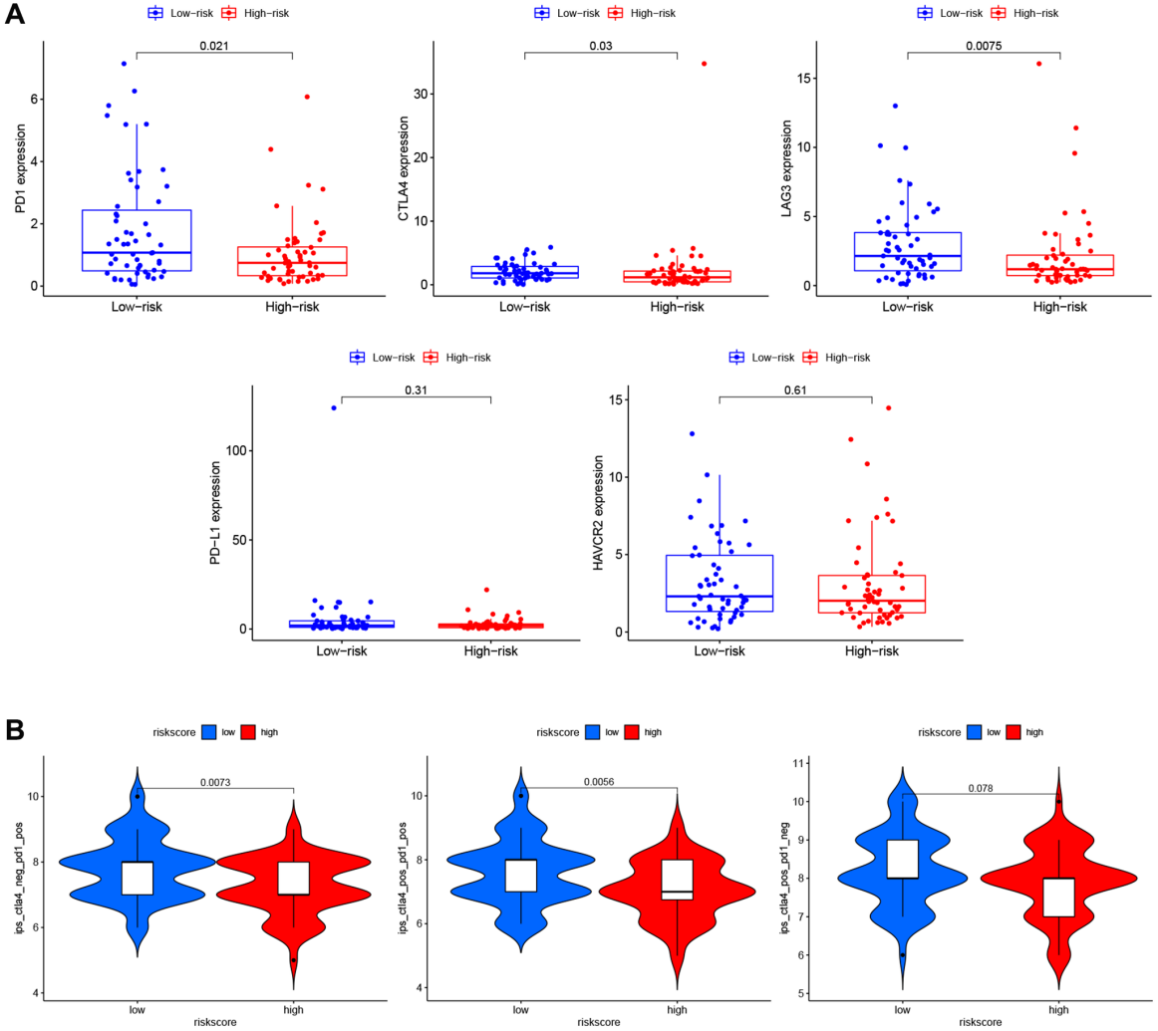
**Immunotherapy prediction of PRGs-based prognostic model for LSCC.** (**A**) Immune checkpoint inhibitors related genes (*PD1, CTLA4, LAG3, PD-L1,* and *HAVCR2*) expression between high- and low-risk patients. (**B**) Differences in immunophenoscores between patients in high- and low-risk groups received anti-PD1 alone, anti-CTLA4 alone, and combination therapy with anti-CTLA4 and anti-PD1.

To explore the role of PRG signature in immunotherapy, we calculated the association of risk scores and the expression of ICI-related genes (*PD1, CTLA4, LAG3, PD-L1,* and *HAVCR2*). The expression of *PD1* (*P* = 0.021), *CTLA4* (*P* = 0.03), and *LAG3* (*P* = 0.0075) were significantly higher in the low-risk group than the high-risk group (Figure 9A), suggesting that the low-risk patients might have a better response to ICIs. We confirmed this finding using TCIA (Figure 9B) and found that the low-risk group had a better response to PD-1 inhibitor alone (*P* = 0.0073) or a combination of PD1 and CTLA4 inhibitor (*P* = 0.0056), but not for the CTLA4 inhibitor alone (*P* = 0.078).

## DISCUSSION

We comprehensively assessed the expression of 47 PRGs in LSCC patients from TCGA and found 25 PRGs that were significantly differentially expressed between tumor samples and adjacent normal tissues; this finding suggests that pyroptosis might play an essential role in the pathogenesis and progression of LSCC. Studies showed that GSDME-expressed cells could be shunted from noninflammatory apoptotic death to inflammatory pyroptotic death [9, 18]. Shao et al. showed that the level of GSDME is the key “switch” for cells towards pyroptosis or apoptosis after caspase-3 activation [18]. In lung cancer, paclitaxel and cisplatin-induced pyroptosis by a caspase-3/GSDME mechanism [19], interestingly, we found significantly increased expression of GSDME, which might act as a risk factor.

To further explore the prognostic value of PRGs in LSCC, we constructed a prognostic PRG signature including six PRGs (*CASP9*, *GSDMA*, *NLRP1*, *IL1B*, *TLR4*, and *IRF8*). *CASP9*, an initiator of intrinsic apoptosis, regulates physiological and pathological cell death in several diseases, including various cancers, neurological disorders, and autoimmune pathologies [20]. *GSDMA*, the earliest identified member of the GSDM family, is located on human chromosome 17q21 [21]. Evidence suggests that intratumoral delivery of nanoparticle-conjugated, pre-cleaved *GSDMA* selectively causes tumor cell pyroptosis due to its N-terminal domains, which forms pores by binding to acidic phospholipids in cell membranes [22, 23]. Human *GSDMA* is expressed in the stomach and skin but is silenced in gastric cancer tissues and cell lines [24]. However, *GSDMA* level could be up-regulated by the DNA methyltransferase inhibitor 5-aza-2’-deoxycytidine (5-aza-dC) in these cells, suggesting that it is suppressed by DNA methylation [25]. *GSDMA* was up-regulated by *TGFβ* and then triggered cell death in the gastric epithelium pit cells, indicating it acts as a tumor suppressor gene in gastric cancer [26]. *NLRP1*, the first human inflammasome sensor, contains leucine-rich repeat and pyrin domain containing 1 [27]. *NLRP1* inflammasomes mediate the production of various cytokines and trigger the inflammatory process [28]. *NLRP1* also participates in the self-destruction of cells, including apoptosis and pyroptosis. Pharmacological inhibition of dipeptidylpeptidase 8 and 9 induced pyroptosis by activating *NLRP1* and *CARD8* [23]. *IL1B*, a member of the cytokine family, participates in inflammation-induced carcinogenesis [29]. It is also known as an alarm cytokine for the response to damage-associated and pathogen-associated molecular patterns by pathogen-recognition receptors and trigger inflammasome activation following cleavage by caspase-1 into its active form of pro-IL-1β [30]. Inflammasome activities cause IL-1β release from living (hyperactive) or dead (pyroptotic) cells depending on the cell type and stimulus [31]. The role of IL-1β in tumors is pleiotropic, including promotion of inflammation-induced carcinogenesis, recruitment of antineoplastic cells, and may block metastatic outgrowth [32, 33]. *TLR4*, a type I transmembrane glycoprotein receptor, was the first discovered TLR in mammals. It is widely expressed in human cells such as mononuclear macrophages and renal tubular epithelial cells [34]. *TLR4* participates in the innate immune response by recognizing lipopolysaccharide, and it also acts as a bridge connecting innate and acquired immunity [35, 36]. Despite studies having reported that *TLR4* is involved in the occurrence and development of liver cancer [37], lung cancer [38], gastric cancer [39], and colorectal cancer [40], the correlation of *TLR4* and LSCC had not been explored. *IRF8* is an interferon regulatory transcription factor family member, also known as interferon consensus sequence-binding protein. It is required for early B cell development with *IRF4* and negatively regulates immune cells [41]. Recently, studies showed *IRF8* was associated with several tumors [42–45]. Taken together, our findings suggest that these six PRGs in the newly established signature have roles in promoting or inhibiting tumor cell pyroptosis, which might provide potential therapeutic targets for LSCC. Kaplan-Meier survival analysis, ROC curve analysis, and multivariate Cox regression subgroup analysis were performed to determine the model's efficiency. We found that the novel PRG signature is a powerful predictor of outcome in LSCC. Calibration plots confirmed that the nomogram incorporating the PRG risk score and clinical risk factors had better predictive accuracy and may be used for risk stratification. In addition, we analyzed the relationship between the clinical factors and risk score, but there was no valuable relevance been observed. We speculated that it was too small quantity of LSCC specimens in each clinical subgroup to explore the relationship of clinical factors and risk score. Future studies with more samples are needed to verify these results.

To explore the biological functions of PRG signature, we conducted GO enrichment analysis based on the DEGs between high- and low-risk groups and found that the DEGs were involved in immune-related biological processes. Further GSEA analysis confirmed that several immune-related pathways were significantly enriched in the low-risk group. There is now substantial evidence that pyroptosis regulates the maturation process of immune cells and immune responses by activating inflammasomes and secretion of inflammatory cytokines [46, 47]. As expected, we observed that the low-risk group had higher immune scores than the high-risk group, with higher infiltration of memory B cells, plasma cells, and CD8+ T cells. CD8+ T cells participate in immune response and producing antitumor response [48, 49]. In contrast, M2 macrophages are immune suppressive cells that might be related to tumor recurrence, metastasis, and poor outcome [50, 51]. In the current study, we found that the content of M2 macrophage cells was higher in the high-risk group and positively correlated with the risk score. We speculate that this is a mechanism explaining better outcomes in the low-risk group. Given our analysis, it is reasonable to conclude that pyroptosis facilitates the recruitment of infiltrating immune cells and regulates the composition of the tumor immune microenvironment to mediate the pathogenesis of LSCC.

Immunotherapy is an essential adjuvant treatment combined with surgery, radiotherapy, and chemotherapy. This therapy induces the immune system to kill tumor cells. This is why immunotherapy might solve the problem of tumor heterogeneity in targeted therapy [52]. The agents of immunotherapy are ICIs that have been helpful in several cancers [53, 54]. Pembrolizumab and nivolumab are approved for treatment o platinum-refractory recurrent or metastatic head and neck squamous cell carcinoma, including LSCC [55, 56]. Of course, many LSCC patients, especially those in the progressive phase, might benefit.

Nevertheless, many patients do not experience good clinical outcomes because of tumor heterogeneity. Therefore, we also compared the expression of ICI-related genes (*PD1, CTLA4, LAG3, PD-L1,* and *HAVCR2*) between the high- and low-risk groups [57–59] and found that *PD1*, *CTLA4,* and *LAG3* were significantly increased in the low-risk group, suggesting that the low-risk patients might have a better response to ICI therapy. We confirmed this using TCIA, finding that the low-risk group had a higher immunophenoscore for PD-1 inhibitor alone or the combination of PD1 and CTLA4 inhibitor. We speculate that our PRG signature might be helpful to develop individualized and precise immunotherapy strategies for LSCC.

This study has some limitations. These results were all based on TCGA, and the number of cases was relatively small for the scarcity of LSCC in the public database. Therefore, we are collecting surgical LSCC tissues for the verification set to detect the level of PRGs and immunological factors in the further study. On the other hand, our finding was lack of mechanisms of PRGs-based prognostic model effect on immunotherapy for LSCC, the conclusions would be more reliable if there were experimental validation. Based on the results of this study, we have conducted LSCC animal models in the new study to explore the correlation of six pyroptosis genes expression and immune cell infiltration.

## CONCLUSIONS

We constructed a novel six-PRG signature for the outcome prediction of LSCC. We indicated PRGs that potentially affect antitumor immunity and may act as immunotherapy targets for LSCC. Our findings provide insight to predict outcomes and identify therapeutic targets for LSCC patients.

## MATERIALS AND METHODS

### Data collection

RNA sequencing data (fragments per kilobase million values) for 111 LSCC tissues and 12 cases of adjacent normal tissue were acquired from TCGA up to 1 July 2021 (https://portal.gdc.cancer.gov/). The corresponding clinicopathological characteristic and prognostic data, including age, gender, histologic grade, T stage, N stage, clinical stage, survival status, and overall survival time, were also downloaded (Table 1). All data are publicly available and followed TCGA data access policies and publication guidelines.

**Table 1:** Clinical characteristics of the LSCC patients in this study.

**Characteristics**	**Number of patients**	**Percent (%)**
Gender		
Female	20	18.02
Male	91	81.98
Age		
<=60	47	42.34
>60	64	57.66
Histologic grade		
G1	8	7.21
G2	70	63.06
G3	29	26.13
Unknow	4	3.6
T Stage		
T1	7	6.31
T2	12	10.81
T3	25	22.52
T4	54	48.65
Unknow	13	11.71
N stage		
N0	39	35.14
N1	12	10.81
N2	39	35.14
N3	2	1.8
Unknow	19	17.12
Clinical stage		
I	2	1.8
II	9	8.11
III	14	12.61
IV	71	63.96
Unknow	15	13.51
Survival status		
Dead	50	45.05
Alive	61	54.95

### PRG expression in LSCC

According to reviews and pyroptosis-related studies [9, 18, 60–74], 47 PRGs were retrieved (Supplementary Table 1). A schematic diagram of the pyroptosis process is shown in Figure 1A. Differential expression analysis of PRGs between LSCC and paracancerous tissues was performed using the “limma” R package. A protein-protein interaction (PPI) network of PRGs was constructed and visualized using the STRING database (https://string-db.org) [75]. The expression correlation network of PRGs in LSCC was also constructed with Pearson correlations > 0.35 and *P* < 0.05.

### Unsupervised clustering analysis

Using the “Consensus Cluster Plus” R package, the mRNA expression profiles of 47 PRGs were adopted for consensus clustering, and the optimal number of clusters (*k* value) was determined according to the resulting cumulative distribution functions [76]. The chi-square test was performed to calculate the association between clinicopathological factors and various clusters.

### Construction of a prognostic PRGs signature

We used univariate Cox analysis to screen the potential PRGs using the “survival” R package with *P* < 0.2. The least absolute shrinkage and selection operator (LASSO) regression method with 10-fold cross-validation was performed to identify the optimal prognostic PRGs for developing the prognostic PRG signature using the “glmnet” R package. The risk scores for all LSCC samples were calculated based on the normalized gene expression levels and corresponding regression coefficients in the model. The formula was as follows: $\text{Risk score}=\sum_{i=0}^{n}\text{Coefficient}\times \text{PRG expression}\text{.}$ Then, 111 LSCC patients were further divided into high- and low-risk groups based on the risk scores' median value. Principal component analysis (PCA) was performed based on expression levels of PRGs in the signature using the “Rtsne” R package. The area under the curve (AUC) values under the receiver operating characteristic (ROC) curve for 1, 3, and 5 years were calculated to estimate the effectiveness of the PRG model using the “survival ROC” R package. The overall survival differences between high- and low-risk groups were compared using Kaplan-Meier survival curves and the log-rank test using the “survminer” and “survival” R packages. Univariate and multivariate Cox proportional hazards regression analyses were performed to determine whether the PRG risk score was an independent prognostic predictor for LSCC. We also developed anomogram for predicting LSCC outcome incorporating age, gender, grade, T stage, N stage, and risk score. The calibration plots were used to assess the prognostic accuracy of the established nomogram. To explore the association between clinicopathological factors and the PRG signature, we performed Chi-square test and Wilcoxon signed-rank test to show the relation between risk score and clinical characteristics including age, gender, tumor grade, clinical stage, T classification, and lymph node metastasis of LSCC patients.

### Gene ontology analysis and gene set enrichment analysis

Based on the differentially expressed genes (DEGs) between the high-and low-risk groups with |log2FC| < 1 and FDR < 0.05, Gene Ontology (GO) analysis was conducted using the “clusterProfiler” R package. Gene set enrichment analysis (GSEA) between the high- and low-risk groups was performed using GSEA 4.0.3.

### Tumor immune microenvironment and immune cell infiltration

The immune score, stromal score, and estimate score of each LSCC patient were calculated using the ESTIMATE algorithm in R software's “estimate” package [77]. The fraction of 22 immune cell types for each sample was calculated using the CIBERSORT algorithm using the “cibersort” package in R [78]. The differences in immune cell infiltration abundances between high- and low-risk groups were illustrated using the “vioplot” package in R. The active scores of 16 infiltrating immune cells and 13 immune-related pathways related were obtained using single-sample gene set enrichment analysis (ssGSEA) with the “GSVA” package in R.

### Association with immunotherapy

We compared expression levels of an immune checkpoint inhibitor (ICI)-related genes (*PD1, PD-L1, CTLA4, LAG3,* and *HAVCR2*) between the high- and low-risk groups. The immunophenoscore of The Cancer Immunome Atlas (TCIA) (https://tcia.at/), which was a superior predictor of response to anti-CTLA4 and anti-PD1 antibodies [78], was applied to predict the potential response of ICI for LSCC patients.

### Statistical analysis

All statistical analysis was performed using R version 4.1.0. The Mann-Whitney U or Chi-square test was used to compare variables. Spearman correlation analysis was used to calculate correlations. Kaplan-Meier analysis with the log-rank test was used to compare survival differences. Univariate and multivariate Cox proportional hazard regression analyses were used to identify independent prognostic factors, and if not explicitly stated, *P* < 0.05 was considered statistically significant. All *P*-values were two-sided.

## Supplementary Materials

Supplementary Figure 1

Supplementary Table 1
