# Supplementary material for: A novel pyroptosis-related gene signature to predict outcomes in laryngeal squamous cell carcinoma
Source: Aging (Albany NY). 2021 Dec 15;13(24):25960–79. doi: 10.18632/aging.203783 (PMC8751611; doi:10.18632/aging.203783)
Supplement: Supplementary Figure 1 [file aging-13-203783-s001.pdf]

SUPPLEMENTARY FIGURE

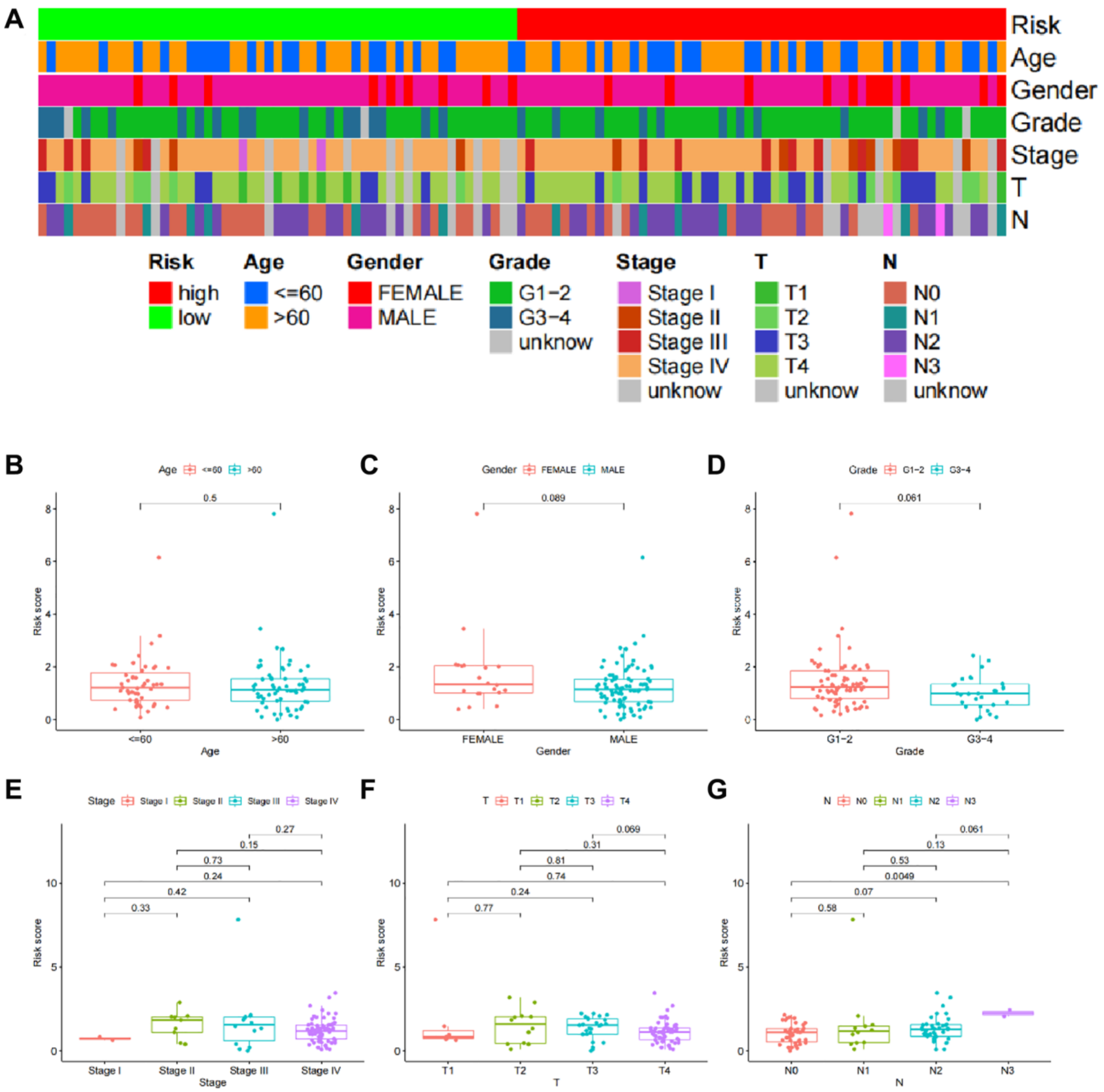

Supplementary Figure 1. Relationship between clinical characteristics and the PRGs-based prognostic model. A heatmap (**A**) and the scatter diagram illustrated age (**B**), gender (**C**), grade (**D**), clinical stage (**E**), T classification (**F**), and lymph node metastasis (**G**) were not linked to the risk score.
