# Supplementary material for: A novel pyroptosis-related gene signature to predict outcomes in laryngeal squamous cell carcinoma
Source: Aging (Albany NY). 2021 Dec 15;13(24):25960–79. doi: 10.18632/aging.203783 (PMC8751611; doi:10.18632/aging.203783)
Supplement: Supplementary Table 1 [file aging-13-203783-s002.pdf]

## SUPPLEMENTARY TABLE

**Supplementary Table 1. 47 pyroptosis-related genes.**

| Genes | Full-names |
|---|---|
| CASP1 | cysteine-aspartic acid protease-1 |
| CASP3 | cysteine-aspartic acid protease-3 |
| CASP4 | cysteine-aspartic acid protease-4 |
| CASP5 | cysteine-aspartic acid protease-5 |
| CASP6 | cysteine-aspartic acid protease-6 |
| CASP7 | cysteine-aspartic acid protease-7 |
| CASP8 | cysteine-aspartic acid protease-8 |
| CASP9 | cysteine-aspartic acid protease-9 |
| GSDMA | gasdermin A |
| GSDMB | gasdermin B |
| GSDMC | gasdermin C |
| GSDMD | gasdermin D |
| GSDME | gasdermin E |
| PJVK | pejvakin/deafness, autosomal recessive 59 |
| NLRC4 | NLR family CARD domain containing 4 |
| NLRP1 | NLR family pyrin domain containing 1 |
| NLRP2 | NLR family pyrin domain containing 2 |
| NLRP3 | NLR family pyrin domain containing 3 |
| NLRP6 | NLR family pyrin domain containing 6 |
| NLRP7 | NLR family pyrin domain containing 7 |
| MEFV | the pyrin inflammasome gene |
| AIM2 | Absent in melanoma 2 |
| NOD1 | nucleotide binding oligomerization domain containing 1 |
| NOD2 | nucleotide binding oligomerization domain containing 2 |
| CARD8 | a caspase recruitment domain |
| PYCARD | PYD and CARD domain containing |
| IL18 | interleukin 18 |
| IL1B | interleukin 1 beta |
| IL6 | interleukin 6 |
| PLCG1 | phospholipase C gamma 1 |
| PRKACA | protein kinase cAMP-activated catalytic subunit alpha |
| SCAF11 | SR-related CTD associated factor 11 |
| TIRAP | TIR domain containing adaptor protein |
| TNF | tumor necrosis factor |
| ELANE | elastase, neutrophil expressed |
| GPX4 | glutathione peroxidase 4 |
| ZBP1 | Z-DNA binding protein 1 |
| GZMA | GranzymeA |
| GZMB | GranzymeB |
| TLR3 | Toll-like receptor 3 |
| TLR4 | Toll-like receptor 4 |
| RIPK1 | receptor interacting protein kinase 1 |
| RIPK3 | receptor interacting protein kinase 3 |
| IRF2 | interferon regulatory factor 2 |
| IRF8 | interferon regulatory factor 8 |
| NFKB1 | nuclear factor of κB |
| NEK7 | kinase NIMA-related kinase 7 |
